# Stereospecific Self-Assembly Processes of Porphyrin-Proline Conjugates: From the Effect of Structural Features and Bulk Solvent Properties to the Application in Stereoselective Sensor Systems

**DOI:** 10.3390/ijms232415587

**Published:** 2022-12-09

**Authors:** Manuela Stefanelli, Gabriele Magna, Corrado Di Natale, Roberto Paolesse, Donato Monti

**Affiliations:** 1 Department of Chemical Science and Technologies, Università di Roma Tor Vergata, Via della Ricerca Scientifica 1, 00133 Rome, Italy; 2 Department of Electronic Engineering, Università di Roma Tor Vergata, Viale del Politecnico 1, 00133 Rome, Italy; 3 Department of Chemistry, Sapienza Università di Roma, Piazzale Aldo Moro 5, 00185 Rome, Italy

**Keywords:** porphyrins, proline, chirality, supramolecular chemistry, sensors

## Abstract

Conjugating the porphyrin ring with an amino acid via amide linkage represents a straightforward way for conferring both amphiphilicity and chirality to the macrocycle. Proline residue is a good choice in this context since its conformational rigidity allows for porphyrin assembling where molecular chirality is efficiently transferred and amplified using properly honed aqueous environments. Herein, we describe the evolution of the studies carried out by our group to achieve chiral systems from some porphyrin-proline derivatives, both in solution and in the solid state. The discussion focuses on some fundamental aspects reflecting on the final molecular architectures obtained, which are related to the nature of the appended group (stereochemistry and charge), the presence of a metal ion coordinated to the porphyrin core and the bulk solvent properties. Indeed, fine-tuning the mentioned parameters enables the achievement of stereospecific structures with distinctive chiroptical and morphological features. Solid films based on these chiral systems were also obtained and their recognition abilities in gaseous and liquid phase are here described.

## 1. Introduction

Supramolecular systems based on porphyrin derivatives are of great importance, owing to the intrinsic conceptual stimuli and their applications in diverse scientific and technological fields such as organic solar cells [1], artificial light-harvesting systems [2,3], catalysis [4] and photocatalysis [5], photodynamic therapy of tumours [6] and sensors [7]. The implementation of elements of chirality [8,9,10] infers to these supramolecular architectures unique properties in terms, for example, of chiral recognition and sensing [11,12], asymmetric catalysis and Circularly Polarised Luminescence (CPL) generation [13,14,15,16].

Several protocols can be pursued for achieving chiral porphyrin-based suprastructures starting, for example, either from achiral macrocycles in the presence of chemical or physical chiral effectors or, alternatively, accomplishing the self-assembly of chiral building blocks in controlled conditions of solvent, temperature, pH or ionic strength. Even if the first approach requires less time-consuming synthetic procedures and strategies [17,18,19,20,21], it may suffer from a lower degree of stereochemical efficiency, being often not detached from relevant stochastic effects or from the presence of adventitious chiral pollutants that are difficult to eliminate [22]. The second approach, which in most cases would circumvent the drawbacks mentioned above, may require arduous and lengthy synthetic strategies and purification protocols [23,24] but undoubtedly offers the noticeable advantage of achieving chiral systems at a high level of stereospecificity, tuned morphology and function [25,26,27,28]. 

**Scheme 1:**
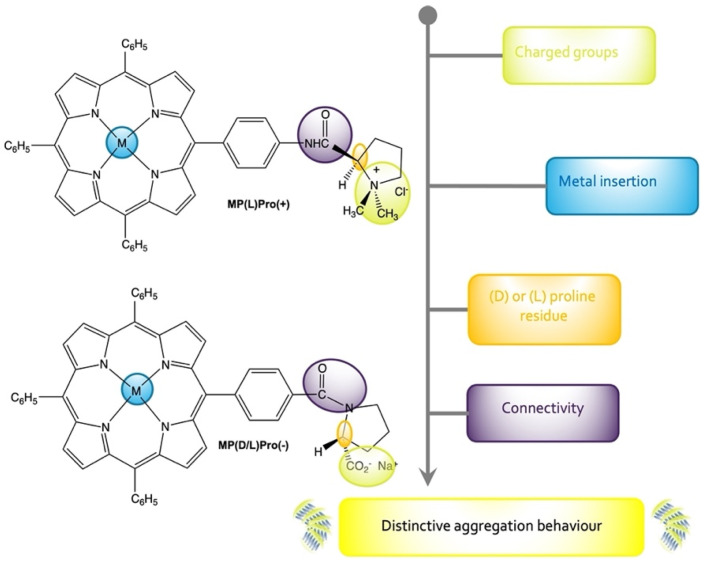
Molecular structures of the porphyrin-proline conjugates herein discussed. The main modifiable molecular synthons on the macrocycles resulting in a distinctive aggregation behaviour are highlighted.

Over the years, we exploited the approach by tailoring the periphery of a tetraphenylporphyrin with a cationic or anionic proline residue (Scheme 1). These moieties have the dual function of both bearing the chiral molecular information and inferring, by virtue of the positive or negative charge present in the group, an amphiphilic character to the whole structure, guiding the self-assembly process by tuning the features of the solvent media. It is well known, in fact, that the solvent properties, in terms of polarity, pH and temperature, may selectively drive the self-recognition attitude of molecular platforms toward the specific formation of the final supramolecular architectures [29]. The enantiomers of proline have been selected as chiral effectors owing to the stereochemical rigid cyclic structure of this amino acid. Proline plays a crucial role in asymmetric organocatalysis with an inherent green shade [30,31,32], and it is also involved in fundamental functions in animal and plant biology [33,34,35]. Very recently, L-proline has been shown to feature “thought-provoking” properties in the chiral resolution of both enantiomers of mandelic acid [36], and the covalent and non-covalent association of self-assembling peptides and tetrapyrrole macrocycles has been reviewed [37].

In this article, we summarise the development of the studies carried out in recent years by our group on the stereospecific self-assembly processes of different amphiphilic porphyrin derivatives (Scheme 1) where the aggregation behaviour and the chiroptical features were finely tuned by means of (i) the metal insertion that reflects on a peculiar coordination ability of the corresponding metalloderivative; (ii) the peripheral charged groups, determining both the solubility and the non-covalent interactions that are established during the aggregation; (iii) the variation of chiral centre configuration, allowing for the achievement of enantiomeric assemblies with specular chirality; and (iv) the different amide connectivity that influences both the stereochemical rigidity of the carboxylate–proline bond, and the nature of the overall intermolecular interactions as a consequence of the opposite charge localised on the stereogenic moiety.

In addition to the structural parameters dealing with the porphyrin ring, we found that the properties of the reaction medium definitely impact the aggregation course, affecting from kinetic aspects to both supramolecular chirality and morphology of the final architectures. The tetrapyrrolic substrates are customarily dissolved as monomers in polar aprotic solvents (i.e., ethanol, THF or acetonitrile), usually in the range of 5 to 10 μM concentration. The self-assembly process is then fostered by the addition of a proper amount of water as a consequence of the onset of hydrophobic effects. This “water-last” protocol ensures optimal reproducibility of the experimental results. It is well known, in fact, that the mixing order of the reactants could severely affect the morphology and, consequently, the physico-chemical features (size distribution, overall chirality) of the obtained supramolecular species [38].

Alongside these systems obtained in solution, porphyrin-proline conjugates “as is” or in combination with inorganic nanoparticles, were also used by our group for the fabrication of chiral solid films that have been exploited in chiral discrimination tests, both in the gas [39] and liquid phase [40], conferring to these systems a practical value in the chiral sensing field.

## 2. Synthesis of the Porphyrin Chiral Derivatives

**Scheme 2:**
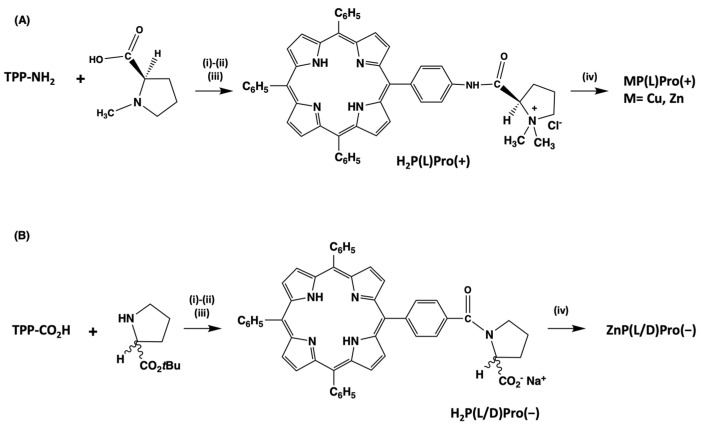
Preparation of the chiral porphyrin derivatives described in this work. (**A**) (i) EDCl, HOBT, dry THF/CH_2_Cl_2_, 0 °C, 1 h, then RT 48 h. (ii) CH_3_I, DMF, RT, 72 h. (iii) NaCl/CH_3_OH, RT, overnight. (iv) M(OAc)_2_, CHCl_3_/CH_3_OH (5/1, v/v), RT, 1 h. (**B**) (i) EDCl, HOBT, dry CH_2_Cl_2_, 0 °C, 1 h, then RT 48 h. (ii) TFA/CH_2_Cl_2_ (2/3, v/v), 1.5 h. (iii) Aqueous NaHCO_3_, then Na_2_SO_4_. (iv) Zn(OAc)_2_, CHCl_3_/CH_3_OH (5/1, v/v), RT, 1 h.

The synthesis of the macrocycles entailed the straightforward coupling of the proper macrocycle with the proline derivative using the well-known peptide coupling reactions. The procedures are summarised in Scheme 2 [41,42,43,44].

The cationic derivative **H_2_P(L)Pro(+)**, for example, is obtained upon coupling reaction from the aminophenyl-porphyrin derivative TPP-NH_2_ with the N-methyl-(L)proline. The subsequent quaternisation of the proline nitrogen with iodomethane gave the desired product. Standard metalation procedures have been followed for obtaining the corresponding metallo-derivatives **ZnP(L)Pro(+)** and **CuP(L)Pro(+)**. The change in the amide connection afforded the anionic porphyrins, starting from the **TPP-CO_2_H** derivative and (L)- or (D)-proline tert-butyl ester that gave the protected macrocycle by analogous reaction conditions. Acid-catalysed removal of the protecting group, followed by standard workup steps including metalation, afforded the desired anionic chiral counterparts **MP(L)Pro(−)** and **MP(D)Pro(−)**, with M = 2H or Zn.

## 3. Overview of Aggregation Behaviour and Kinetics of Porphyrin Derivatives

**Scheme 3:**
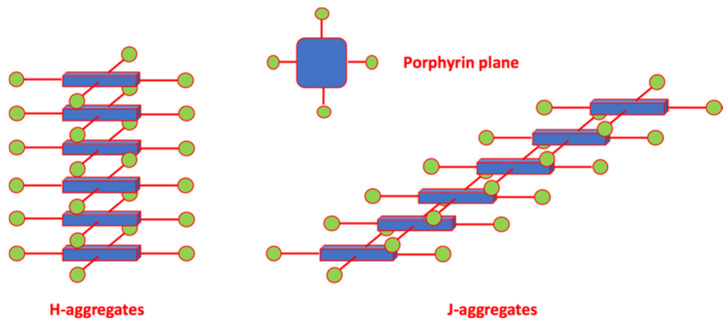
Idealised cartoon of the limiting molecular morphologies of H (**left**) and J (**right**) porphyrin aggregates.

Aggregation is a phenomenon of reversible association of monomers bound by non-covalent interactions including: hydrogen bonds, electrostatic or dipolar interactions, π-π interactions and London dispersion forces. The most common geometry for porphyrin aggregates is the staggered π-stacking, in which an electron-rich pyrrole ring overlaps the central electron-deficient cavity of the adjacent molecule. Since the aggregates can be dimers, trimers, oligomers or aggregates of a higher order, their electronic spectra exhibit a rich florilegium of complex patterns. The spectral variations with respect to the monomeric form, such as the decrease in intensity of the Soret band, its broadening and the shift in wavelength maxima, still provide useful information to describe the morphology of the supramolecular structures. The most well-known aggregate forms are those of type J and H, after E.E. Jelley and the term hypsochromic, respectively [45,46]. The distinction between these two forms is based on the dipole moment orientation of chromophores, resulting in a different shift of the Soret band. H-type aggregates present the stacked chromophores with face-to-face orientation, in which the maximum overlay of type π-π results in blue-shifted Soret bands (Scheme 3).

The first explanation of the shift of λ_max_ was provided by Kasha, whose model still represents a useful qualitative approach, although it has been successively modified. The excitonic coupling theory describes the interaction between the transition moments of the chromophores, and the consequent energetic splitting of the excited level, depending on their geometric arrangement, according to the approximation of the point dipole [47]. J-type aggregates are generally characterised by side-to-side chromophores with an oblique and offset overlap; a type J arrangement was observed with both homo- and hetero-aggregates of amphiphile porphyrins [48].

The kinetic study of the aggregation reactions of these macrocycles plays a fundamental role. Often the mechanism with which the phenomenon takes place has a determining role in the morphology and the physico-chemical and stereochemical properties of the final macroscopic structures. The growth of aggregates of porphyrins can be easily followed over time by spectroscopic techniques such as UV-Vis, Circular Dichroism, Fluorescence and Resonance Light Scattering. There are two most common kinetic pathways, each reflecting in a different type of aggregation mechanism that will therefore be considered separately. It should be remembered, however, that crossover between different kinetic decays can be observed depending on the reaction conditions and structural features of the macrocycles.

### 3.1. Diffusion Limited Aggregation (DLA)

This type of kinetic profile reflects a mechanism that provides for an initial formation of moderately sized clusters, followed by their growth by incorporating the remaining monomers in solution. The kinetic law that adheres to the experimental points of this decay is as follows:

(1) $$Y=Y_{0}+\left(Y_{\infty }-Y_{0}\right)\left[1-e^{-{\left(kt\right)}^{n}}\right]$$

In this equation *Y* represents the physicochemical properties of the system (molar concentration, molar extinction, fluorescence emission intensity and so on), k represents the apparent first-order constant, while the parameter “*n*” is an indicator of the growth factor of the aggregates according to a mechanism limited by diffusion, which in the literature takes the name of DLA (Diffusion-Limited Aggregation). Stretched exponentials are characterised by an exponent n<1. When n=1, the equation reduces to the usual exponential distribution process usually called RLA (Reaction-Limited Aggregation), which corresponds to a first-order process. The term stretched reflects the fact that the relaxation is slower than a purely exponential dependence on time. Examples of DLA behaviour have been thoroughly studied in the past by Monsù Scolaro and co-workers [49,50,51].

A related mechanism, i.e., the Diffusion Limited Cluster–Cluster Aggregation (DLCCA) is also encountered [52], and can be distinguished by the former DLA by examining the fractal, or Hausdorff dimension *d_f_*, that can be derived from scattering techniques [49].

### 3.2. Autocatalytic Kinetic Profile

This behaviour, characterised by a typical sigmoidal shape, was first observed by Robert F. Pasternak and co-workers in their seminal kinetic studies of the formation of porphyrin aggregates on DNA [53]. This profile is typical of autocatalytic processes, which lead to the development of species with peculiar fractal morphology. In nature, this phenomenon can be commonly observed, for example, in the formation of dendrites of transition metals, their oxides or ice crystals and so on. This mechanism is characterised by an initial slow stage (lag phase), in which nucleation seeds are formed, allowing a subsequent catalysed growth of the aggregates. The presence of this phenomenon is justified by the fact that the aggregation nuclei offer a more extensive interaction (wider non-covalent surface), catalysing the growth of supramolecular systems. The integrated kinetic equation is:

(2) $$\left[M\right]={\left[M\right]}_{\infty }+\left({\left[M\right]}_{0}-{\left[M\right]}_{\infty }\right)\left\{1+\left(m-1\right){\left[k_{0}t+{\left(n+1\right)}^{-1}{\left(k_{cat}t\right)}^{n+1}\right]}^{-\frac{1}{m-1}}\;\right\}$$

where [*M*] is the concentration (or other physical quantity related to it such as, for example, absorbance, CD or fluorescence emission intensities) at time “*t*”, [*M*]_0_ and [*M*]_∞_ are the concentrations at the start (*t* = 0) and the end of the process (*t* = ∞) and parameter “*m*” estimates the size of the nucleating centres, indicating the number of monomers that constitute a growth centre acting as a surface for the catalysed growth. The parameter “*n*” indicates instead the degree of cooperativity of the process and strictly influences the speed of the global assembling. The term $k_{0}$ indicates the kinetic constant of the uncatalysed parallel process, in which the monomer interacts with other single monomers, or with nuclei characterised by an aggregation number lower than m. Finally, *k_cat_* indicates the catalysed rate constants of the growth of structures on suitably sized nuclei. This equation is valid for m>1 and both $k_{0}$ and *k_cat_* have the dimensions of $s^{-1}$ and for t→0 the constant *k_cat_* → *k*_0_. In some instances, the values of *k_cat_* could show a dependence on the initial concentrations of the solutions, indicating the formation of “nucleation seeds” of different nature and morphology.

This nonconventional approach has been successfully employed for the investigation of growth processes of porphyrin aggregates on DNA matrices and other chiral polymers, for different and important biological self-assembled systems [54] and in acid-catalysed water-soluble porphyrin aggregation [55].

A particular form of this equation has been developed by Pasternack, for the case of disassembly of a DNA-bound porphyrin aggregate by β-cyclodextrin [56].

(3) $$\left[M\right]={\left[M\right]}_{\infty }+\left({\left[M\right]}_{0}-{\left[M\right]}_{\infty }\right)exp\left\{-{\left(k_{f}t\right)}^{n+1}/\left(n+1\right)\right\}$$

This is a reduced form of the Equation (2), at the limiting value of *m* → 1, indicating that any monomer itself constitutes a catalysed nucleation centre. The parameter *k_f_* is the rate constant of formation of aggregates in s^−1^ units, and “*n*” is again the cooperativity factor of the formation process. Notably, in the case of *n* = 0, the equation assumes the form of a conventional first order kinetic law.

## 4. Aggregation Behaviour and Supramolecular Chirogenesis of the Free-Base Derivatives

**Figure 1:**
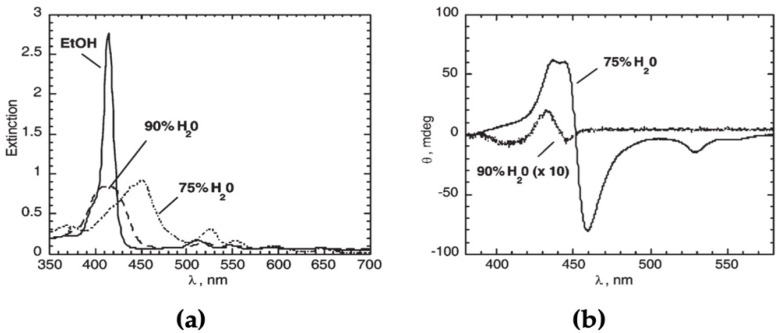
(**a**) UV-Visible spectra of the aggregates of **H_2_P(L)Pro(+)** in different hydroalcoholic media; the spectrum in non-aggregative conditions (pure ethanol) is reported for comparison. (**b**) Comparison of the CD spectra of the aggregates obtained from different water-containing solvent mixtures. Adapted from [41], with permission from the Royal Society of Chemistry, Copyright © 2005.

Initial studies carried out on the cationic free-base **H_2_P(L)Pro(+)** put in evidence the fundamental aggregation features of this kind of substrate, with reference to the nature and the composition of the solvent [41]. The title porphyrin is in monomeric form in ethanol, while in 75/25 v:v hydroalcoholic medium, the species is prone to self-assemble. The formation of aggregates is evidenced by the typical changes in the spectroscopic pattern of the macrocycles that presents a bathochromic effect and lowering of the intensity of the main Soret B band (Figure 1a). Notably, the redshift evidences the formation of supramolecular species with local offset, J-type topology. This proper solvent composition allows for an optimal reaction rate that can be followed by conventional spectroscopic means (UV-Visible), revealing an autocatalytic behaviour typical of the templated formation of supramolecular fractal structures in the case of water soluble tetrapyrrolic platforms [53]. Worthwhile to note, the aggregation proceeds with the formation of J-type, offset structures featuring intense supramolecular chirality as a consequence of the reading-out and transfer of the chiral information stored on the proline cationic stereogenic centre (Figure 1b).

Conversely, in a medium constituted by a higher amount of water (90/10 v:v) in which the hydrophobic effect is much more impelling, the aggregation process is faster and the final species are characterised by a lower degree of supramolecular chirality, as shown by the corresponding CD spectra of weaker intensity and the apparent no shift of the Soret band in the UV-Vis spectra (Figure 1a,b). Corresponding Resonance Light Scattering (RLS) studies indicated that the slower and more specific self-assembly process leads to the formation of ordered structures with more effective electronic coupling among the aromatic platforms. RLS is a powerful spectroscopic technique developed in the past by Robert Pasternack to study porphyrin aggregates, consisting of increasing the intensity of the scattered light in the proximity of the electronic absorption of the aggregates, typically at the Soret B band [57,58]. RLS intensity is related to the number, size, and coherence length of the aggregates, i.e., the extent of the electronic communication among the aromatic platforms [59].

**Figure 2:**
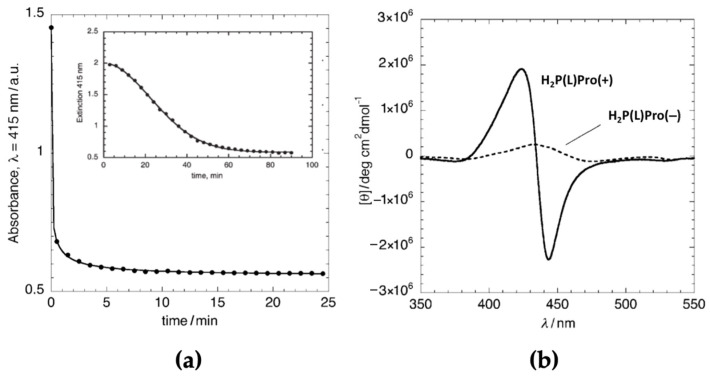
(**a**) Kinetic profile (UV-Vis intensity variation of Soret band) of **H_2_P(L)Pro(–)** in EtOH/H_2_O 25/75 v:v. The inset reports the corresponding profile for the **H_2_P(L)Pro(+)** counterpart. (**b**) Comparison of the CD profiles of the porphyrin-free base derivatives. Adapted from [43], with permission from Wiley-VCH, Copyright © 2010, and from [41], with permission from the Royal Society of Chemistry, Copyright © 2005.

Analogous studies carried out on the anionic counterpart **H_2_P(L)Pro(−)** [43] showed different kinetic behaviour in the same hydroalcoholic solvent mixture, featuring a much faster DLA process (Figure 2a). This difference reflected itself in the supramolecular chirality of the final architectures, with the formation of species with less intense dichroic features of about one order of magnitude (Figure 2b), being [θ] = 2 × 10^6^ and 3 × 10^5^ deg cm^2^ dmol^−1^ for the cationic and the anionic derivatives, respectively, at 5.0 μM concentration.

Analogous findings have been reported by Monsù Scolaro, who showed that the kinetic of the aggregation strongly influences the chirality of the growing species [60]. A recent work of Meijer and co-workers highlighted the effect of the “C=O centred” vs. the “NH centred” motifs of the amide connectivity of a porphyrin macrocycle with an uncharged stereodirecting group, due to their different strengths of molecular interaction and conformational flexibility [61]. The macrocycle with the C=O centred motif yielded H-type aggregates with intense supramolecular chirality, conversely to the NH centred counterpart that formed achiral J-type species. In our case, an opposite trend is found since the “NH-connected” cationic platform leads to the formation of suprastructures with the most intense chirality. Hence, the different behaviour observed should be inferred to the nature of the charges residing on the proline functionality that modulates the physicochemical behaviour of the macrocycles and the interactions onset during the supramolecular polymerization.

However, it must be emphasised that disregarding the nature of the charge of the proline residues, the same J-type aggregates with the same −/+ signs are observed for the CD bands of both types of supramolecular species, indicating the same anti-clockwise porphyrin-to-porphyrin spatial arrangements, dictated by the configuration of the chiral L-proline moieties [62].

**Figure 3:**
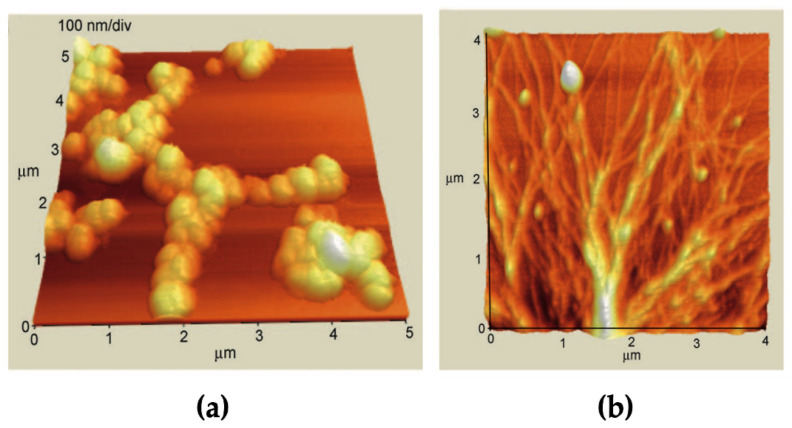
AFM images of porphyrin aggregates layered onto HOPG, formed in EtOH/H_2_O 25/75 v:v. (**a**) **H_2_P(L)Pro(−)**; (**b**) **H_2_P(L)Pro(+)**. Adapted from [43], with permission from Wiley-VCH, Copyright © 2010.

Atomic Force Microscopy topographic imaging of the cationic and anionic aggregates on HOPG revealed important differences also on the morphology of these structures, showing a globular appearance for the anionic derivatives and entangled fibrillar structures for the cationic counterpart (Figure 3) [43].

A remarkable aspect of the above-described systems is that the anionic aggregates of **H_2_P(L)Pro(−)**, act as an efficient substrate for the templated aggregation of the cationic analogue **H_2_P(L)Pro(+)**, resulting in the final “catanionic” species with increased both supramolecular chirality and the aggregation rate by about one order of magnitude [63].

Interestingly, the opposite experiment, i.e., templated aggregation of the negative macrocycles onto cationic aggregates, did not amplify the final chiroptical features, suggesting that the process complies with the so-called sergeant-soldier principle. This effect could be ascribed to the diverse degree of hydrophilicity/lipophilicity of the charged groups, i.e., quaternary ammonium and carboxylate moieties, that onset peculiar competitive intermolecular interactions such as π-cation or hydrogen bond with the N or NH groups of the inner cores of the aromatic platforms and solvation forces with the bulk solvent. According to the Hofmeister series [64], the carboxylic groups are characterised by higher hydrophilicity and tend to be more exposed to the external side of the aggregates exerting a more effective electrostatic interaction toward the incoming cationic ammonium groups. On the other hand, these latter, more polarisable, species are more prone to interact with the inner aromatic part, resulting in a less effective interaction with the incoming carboxylate moieties. This subtle interplay of electrostatic and hydrophobic effects has been more recently rationalised by Monsù Scolaro, who showed a clear dependence on the nature of the counter-anion on both the kinetics and the final overall chirality of water-soluble achiral porphyrin aggregates (TPPS_4_) catalysed by inorganic acids [64]. The reactivity order (i.e., the extent of conversion and the kinetic constant values) are in the order H_2_SO_4_ > HCl > HBr > HNO_3_ > HClO_4_, from the “least-perturbing” anion to the “most-perturbing” one, with respect to the hydrogen-bond network of the solvent. Worthwhile to note, the dissymmetry factor (g) of the resulting CD spectra shows an opposite trend, being the highest in the case of the perchlorate anion. Interestingly, in all of the cases the CD spectra feature a spectral pattern of positive Cotton effect, although a random statistical outcome would be expected, and is commonly observed, for a symmetry-breaking event on an achiral substrate [65,66]. However, this preferential chiral induction is not unprecedented, although it is of still unclear origin [22,67]. Moreover, it has been reported in a case, that the templated formation of chiral J-aggregates both the kinetic and the final anisotropy factor of the formed species are dependent on the enantiomer employed as a chiral effector [68].

## 5. Aggregation Behaviour and Supramolecular Chirogenesis of the Metallo-Derivatives

### 5.1. Aggregation Behaviour of Cationic **MP(L)Pro(+)**

The coordination of a divalent cation within the tetrapyrrolic core resulted in different aggregation kinetic and chiroptical features of the final supramolecular species.

**Figure 4:**
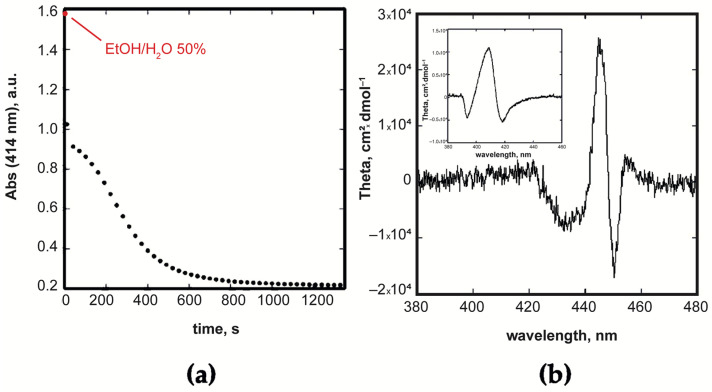
(**a**) Kinetic course for the aggregation of **CuP(L)Pro(+)** showing the occurrence of an initial burst followed by a slow autocatalytic step. The initial point (t_0_) is calculated in non-aggregative conditions (50 % hydroalcoholic medium). (**b**) CD plot for the aggregates of **ZnP(L)Pro(+)**; in the inset is reported the analogous spectrum for the **CuP(L)Pro(+)** derivative. Adapted from [69], with permission from the Royal Society of Chemistry, Copyright © 2019.

In both of the cases (M=Zn(II) or Cu(II) porphyrins), a distinct biphasic process is observed [69] (Figure 4a), consisting in a fast non-specific “initial burst” occurring within the time of preparation of the solution, followed by a slower step of structural rearrangement of the initially formed non-coherent CD silent structures, which give rise to the formation of chiral supramolecular species.

In the case of the Zn(II) complex in the same 25/75 EtOH/H_2_O v:v medium, the second slower step is characterised by an apparent first-order kinetic. The final chiral assemblies feature absorption spectra with a peculiar, coupled pattern for the Soret band that reflected itself in double-bisignated Cotton bands in the CD spectra. These bands have different intensities of −/+ sign that are indicative of an anti-clockwise mutual disposition of the porphyrin platforms [62] (Figure 4b).

The overall CD intensity is about two orders of magnitude lower than that observed in the free-base counterpart, indicating a strong coordination effect to the central Zn ion by the solvent that hampers the formation of a structure in which the macrocycles are coupled at a close distance. The striking role played by the reaction medium is also witnessed by the fact that in dimethylacetamide/water, a strongly coordinating solvent, aggregates no longer feature any supramolecular chirality.

In the case of the Cu(II) derivative, a similarly coupled Soret band and corresponding bisignated negative CD spectra are obtained (Figure 4b, inset). Remarkably, different kinetic behaviour for the second process is found, following a cooperative autocatalytic path in this case. This mechanistic difference can be attributed to the lower tendency of the tetra-coordinated metal centre to onset further interaction with the solvent.

### 5.2. Through the Looking Glass: Aggregation Behaviour of Anionic **MP(L)Pro(−)** and **MP(D)Pro(−)** (M = 2H or Zn)

The results obtained in the solvent-driven aggregation of the L- and D-enantiomer of the Zn-anionic porphyrin confirmed the stereospecificity of the molecular recognition that strictly follows the chiral information stored on the anionic proline group. In particular, the (L)-derivative gives rise to the formation of chiral J-type structures featuring a CD spectral pattern governed by an intense coupled, bisignated band of positive sign ([*θ*] = 10^7^ deg cm^2^ dmol^−1^), indicating a mutual clockwise arrangement of the macrocycles. The other enantiomer behaves in specular mode, ending with the formation of anti-clockwise structures [44]. Interestingly, a closer look at the CD spectra clearly revealed in both cases the presence of a coupled band at 440 nm and a more diffused one at 418 nm, in close correspondence with the UV-Vis spectra. This peculiar pattern has been ascribed to the formation of J aggregates of complex morphology, with excitonic coupling along two different space directions [70]. A notable example has been quite recently reported by Short and Balaban, who demonstrated the complex morphology of water-soluble achiral porphyrin J aggregates in the form of intrinsically chiral helical structures arranged in nanotube shape [71].

**Figure 5:**
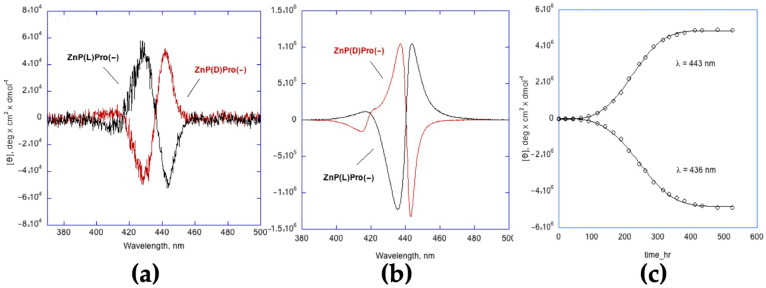
Aggregation of **ZnP(L)Pro(−)** and **ZnP(D)Pro(−)** (5 μM; EtOH/H_2_O 25/75 v:v). (**a**) CD plot at the initial (t = 0) stage; (**b**) CD plot at equilibrium. (**c**) Autocatalytic kinetic profile for the second stage of the aggregation of the (L)-derivative. The points represent the variation of the CD bands vs. time at the given wavelength. Adapted from OPEN ACCESS [44].

In our case, detailed kinetic studies revealed a complex reaction pathway consisting of first, a rapid formation of species characterised by a low supramolecular chirality ([*θ*] = 10^4^ deg cm^2^ dmol^−1^) that evolves through a slow autocatalytic process toward the formation of the final highly chiral structures (Figure 5b). Interestingly, the best fit of the data (obtained by either UV-Vis or CD spectroscopy; Figure 5c) is accomplished by using the Equation (3), indicating that the initially formed structures would act as a “single-site” enantiospecific surface (i.e., a collection of independently catalytic chiral sites with the same reactivity).

The different striking behaviour of the anionic Zn-derivative with respect to the free-base analogue is certainly attributable to the intermolecular coordination of the prolinate effector to the inner Zn(II) ion, accounting also for the high intensities of the chiroptical features of the corresponding structures. The involvement of the Zn-coordination is also demonstrated by the results obtained in the aggregation experiments carried out on the free-bases **H_2_P(L)Pro(−)** and **H_2_P(D)Pro(−)**, which form chiral aggregates with CD bands of two order of magnitude lower in intensities and with opposite signs, with respect to the corresponding Zn-derivatives. Moreover, a recent account demonstrated the involvement of Zn-coordination in the formation of porphyrin’s supramolecular polymerisation [72].

**Figure 6:**
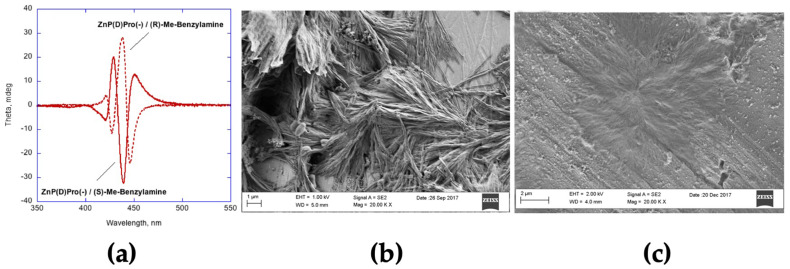
(**a**) CD spectra of **ZnP(D)Pro(−)** aggregates (5 μM; EtOH/H_2_O 25/75 v:v) in the presence of (R)-phenylethylamine (dotted line) and (S)-phenylethylamine (continuous line). SEM images of **ZnP(L)Pro(−)** aggregates (10 μM; EtOH/H_2_O 25/75 v:v) in the presence of (R)-phenylethylamine (1 mM) (**b**) and without added nitrogen ligand (**c**). Adapted from OPEN ACCESS [73].

Moreover, we found that the presence of 100-fold molar excess of external nitrogen ligands, i.e., (R)- and (S)-1-phenylethylamine, dictated the stereochemical course of the self-assembly process, disregarding the stereochemistry of the L- or D-proline functionalities (Figure 6a) [73]. Furthermore, the species formed using an achiral ligand (i.e., benzylamine) no longer showed supramolecular chirality. Finally, the presence of the chiral amines has been found to remarkably impact the morphology of the aggregates, appearing as bundles of rod-like structures, differently to those of fractal shapes formed in bare aqueous solvent mixtures, without nitrogen ligands (Figure 6b,c).

## 6. Transfer of Supramolecular Chirality from Solution to Solid State: Chiral Films on the Glass Surface

The transfer of chiral porphyrin aggregates and related structures on solid surfaces is an issue of great importance for the construction of advanced materials endowed with specific properties and functions, which strongly depend not only on the intrinsic molecular structure but also on their supramolecular organisation [74]. This section describes the development of the construction of solid-state films composed of the title macrocycles.

### 6.1. Fabrication of Solid Films of Cationic Porphyrin Derivatives **MP(L)Pro(+)** (M = 2H, Zn, Cu)

Aggregates of the cationic porphyrins show the peculiar tendency to spontaneously layer onto solid substrates, such as glass or quartz slides, from hydroalcoholic solutions. The thickness of the obtained film, measured by the optical density of the solid layers, depends on the initial concentration of the solutions. In the case of achiral cationic analogs, the spontaneous deposition been exploited for the fabrication of solid-state sensors for Hg^2+^ ions in water [75] and the assessment of food quality in real matrices [76]. The same procedure has been employed for the construction of porphyrin-silica nanoparticles conjugates [77] and a selective ionophore in PVC-membrane sensors [78].

**Figure 7:**
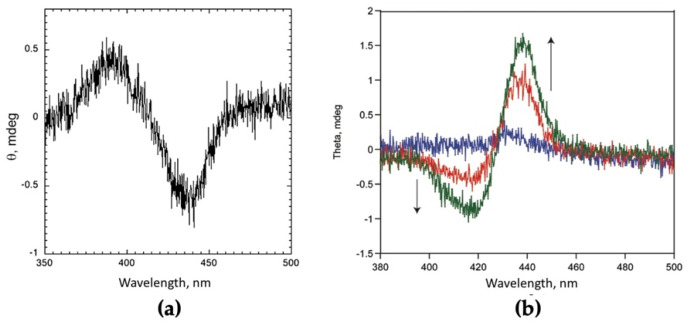
(**a**) CD spectrum of a spontaneously deposited layer of **H_2_P(L)Pro(+)** aggregates from EtOH/H_2_O 25/75 v:v. (**b**) CD spectra of LB films of **H_2_P(L)Pro(+)** deposited on quartz in an increasing number of layers (blue trace, monolayer; red trace, 5 layers; green trace, 7 layers). Adapted from [42] and from [79] with permission from the World Scientific, Copyright © 2011 and © 2019, respectively.

In the case of the present chiral cationic derivatives, the spontaneous deposition yielded to porphyrin films with retained supramolecular chirality (Figure 7a) [42]. The same substrates, once transferred onto quartz slides by means of Langmuir–Blodgett technique, still formed chiral films but, in the case of the cationic free-base (M = 2H), of opposed chirality with respect to that of the films obtained upon spontaneous deposition in equilibrium conditions (Figure 7b) [79].

This finding, important for selecting the overall chirality of the deposited material, has been inferred to the effect of the physical forces applied during the formation of the compressed porphyrin layer at the air–water interface.

### 6.2. Fabrication of Solid Films of Anionic Porphyrin Derivatives **ZnP(L)Pro(−)** and **ZnP(D)Pro(−)**

Differently from the cationic congeners, the anionic derivatives do not spontaneously layer onto dipped solid substrates. However, their solid-state films could be conveniently prepared by the drop-casting method, an easy and versatile means for obtaining functionalised surfaces whose properties feature satisfactory reproducibility. As far as the chirality transfer is concerned, the successful outcome depends on a subtle interplay of factors such as, among others, solvent bulk properties (i.e., viscosity, vapour pressure) and the interaction of the molecular substrates with the surface [80,81].

Our recent studies pointed out that the presence of oligo-aggregates of porphyrin in the bulk solution, strictly related to the solubility of the macrocycle and its concentration, is one of the important issues in forming a solid film with supramolecular chirality. Indeed, these systems act as chiral molecular seeds during solvent evaporation, allowing the following self-assembling process into larger structures [82]. The roughness of the receiving surface has also been found to play a determinant role. Excellent results in terms of reproducibility and overall chirality were obtained, for example, in the case of the enantiomers of the Zn-derivatives from toluene solutions of 10^−4^ M concentration on ultra-flat quartz-coated glass slides (i.e., with the roughness of ca. 1 nm) [83]. Indeed, this aromatic solvent, besides the noncoordinating character, possesses the optimal physical properties in terms of surface tension and vapor pressure that ensure the film adhesion to the substrate and prevent a too fast solvent evaporation. These two factors are essential for the stereospecific organisation of porphyrin macrocycles onto the glass.

## 7. Development of Sensors Based on Chiral Solid Films of Porphyrins MP(L)Pro(−) (M = 2H, Zn)

**Figure 8:**
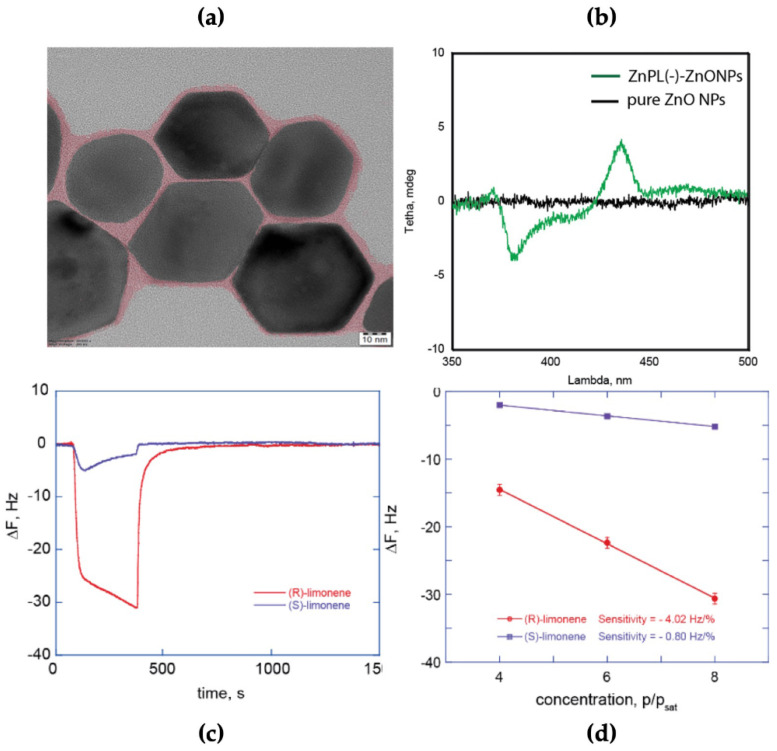
(**a**) TEM images of pyramidal ZnO nanoparticles capped by a layer of **ZnP(L)Pro(−)** aggregates (artificially shown as a pink colour). (**b**) CD spectra of drop-casted material; black-trace, bare ZnO nanoparticles; red-trace, ZnO nanoparticles-porphyrin aggregates conjugates, evidencing the induced chiral feature of the zinc oxide surface (ca 375 nm). (**c**) Dynamic response of a QMB device with a layer of ZnO nanoparticles-porphyrin aggregates conjugates upon exposure to vapours of (R)-limonene (red-trace) and (S)-limonene (black-trace). (**d**) Plot of QMB responses at the different dilutions of the saturated vapour pressure of (R)-limonene (red trace) and (S)-limonene (black trace). Adapted with permission from [39], Copyright © 2019, American Chemical Society.

Chiral layers of the anionic derivatives containing the (L)-Proline group have been deposited on different surfaces and tested for the recognition of couples of analyte enantiomers. Stereoselective sensors were, for example, developed by anchoring **Zn(L)Pro(−)** to ZnO nanoparticles. This hybrid material possesses a combination of unique properties such as (i) an increased surface area and (ii) a low thickness of the sensing film, allowing stereoselective detection also in a high concentration range of chiral analytes. As shown by TEM images (Figure 8a), the porphyrins act as a glue connecting different nanoparticles in the resulting film. Remarkably, the solid-state films showed interesting chiroptical activity as evidenced by electronic circular dichroism (ECD) that highlighted an induction of chirality from porphyrins to ZnO NPs (a dichroic band appears in the UV region), due to electronic coupling existing between the two components (Figure 8b).

An easy way to fabricate a chemical sensor consisted of drop-casting this hybrid material onto a mass transducer such as a quartz microbalance (QMB). So-made sensors displayed stereoselective recognition properties for the (R)-limonene enantiomer vapours (Figure 8c,d) [39]. The high sensitivity featured toward limonene vapours, compared to other gas-phase chiral analytes tested as α-pinenes or butan-2-ols, is attributable to the ease of intercalation of the flat molecules within the porphyrin platforms and on the capability to onset π−π interactions.

**Figure 9:**
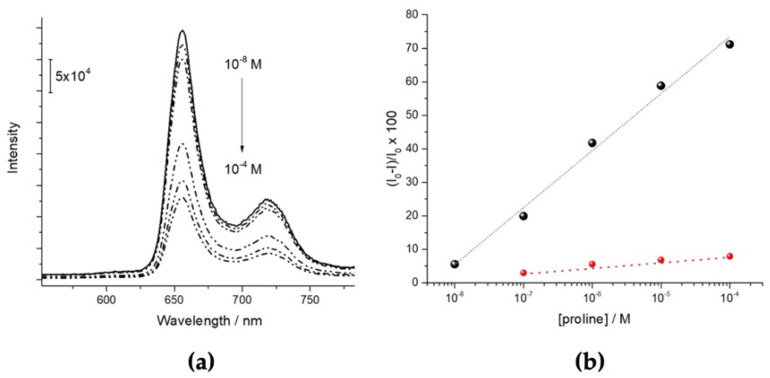
(**a**) Fluorescence quenching of **H_2_P(L)Pro(−)** LS film in the presence upon exposure to aqueous solutions of (L)-proline at increasing concentrations. (**b**) Quenching of fluorescence of **H_2_P(L)Pro(−)** LS film in the presence of (L)-proline (black dots) and (D)-proline (red dots). Adapted from OPEN ACCESS [40].

Very recently, we found that Langmuir–Shaefer films of the free-base derivative **H_2_P(L)Pro(−)** can selectively recognise the same L-amino acid from the D-enantiomer in the water solution [40]. In this case, the deposition process seems to promote the porphyrins transfer as monomers on the substrate, allowing the selective interaction with the fluxed L-proline. The binding with the amino acid induces aggregation and a consequent fluorescent quenching that can be used as an analytical signal (Figure 9a). The same effect is only marginally observed under fluxing D-Proline solutions (Figure 9b), demonstrating the enantioselectivity of the system.

This interesting finding would pave the road for the construction of tailored solid-state sensible material with selective chiral recognition features that can be specifically tailored for a given enantiomer.

## 8. Conclusions and Future Perspectives

In this work, the aggregation properties of amphiphilic porphyrin derivatives bearing an ionic proline functionality have been summarised, pointing out the effect of the charge’s nature and the chirality of the appended chiral effectors and of the bulk solvent properties on the stereospecificity of the self-assembly processes. The possibility of transferring the chiral features in the solid state has been also discussed, together with the eventual application of the obtained material for the stereoselective detection of chiral vapors by piezoelectric mass transducers (QMB-electronic nose).

In the light of the possibility of extending the arena of the involved macrocycles by further functionalisation of both their periphery and their inner core, the presented results are of importance for the development of systems implemented in sensors arrays [84], are able to discriminate a large class of analytes for the monitoring of food quality [85], as well as environmental and health safety. According to the data reported in a recent review, in fact, ca. 40% of the likely underestimated 4000 environmental pollutants are chiral [86]. These species, caused by their continuous release and accumulation in the environment (soils, freshwaters and biota) unavoidably enter the food chains, resulting in severe effects for human and other living species [87,88,89,90]. A new emergent protagonist in the theatre of the methods developed for facing this highly impactful problem is graphene and its derivative graphene oxide (GO) [91], which have been demonstrated to show promising performances as absorption and remediation agents in wastewater treatment plants [92]. The implementation of elements of chirality, for example, in GO derivatives would represent a fine tuning of the above-described properties for the stereoselective detection and removal of chiral noxious wastes. The chiral functionalisation of GO surfaces has been reported, mainly through bonds with amino acid derivatives [93,94,95]. Although both non-covalent and covalent porphyrin-GO conjugates are widely reported [96], mostly for photovoltaic [97] or cancer treatment [98,99,100,101], none of which involve *strictu sensu* chiral porphyrins, except for the presence of a coordinated chiral group to the central metal ion [102,103].

Within this respect, we can surmise that the incorporation of our restless proline-porphyrin derivatives onto the GO surface would represent a step forward to fulfill the need of systems for selective recognition and removal of the above-cited pollutants. Studies on this interesting topic are actively being carried out in our groups, and the results will be reported in future.

## Data Availability

Not applicable.

## Dedication

This work is dedicated to the memory of Professor Robert F. Pasternack, for his ground-breaking contribution to the chemistry of porphyrin aggregates.
